# Editorial Note

**Published:** 1950-07

**Authors:** 


### Editorial Note

**To Account rendered**

How pleasant to feel that anyone who is sick, be the malady trivial or serious, can obtain the best possible medical care, without any worry about the expense to add to his distress. That the expense to the community has already become stupendous, since this once enviable privilege became the legal right of all, is no matter for surprise. What *is* astonishing is that our " statesmen ", our grave leaders, our omniscient planners apparently never even suspected such a result!

Their reaction has been brisk. (*Dare* we couple Mr. Bevan and " reaction "?) " It is necessary to call a halt to further development of these services."* Every Regional Board has had peremptory orders to reduce substantially its estimates for 1950 and to keep strictly within the reduced total. Henceforward the test is not " Is this desirable? " but, " Is this essential and can we afford it? "—much as before the halcyon days of " planning". Even so, we shall this year spend on the treatment of disease well over £10 for every man, woman and child in the kingdom, nearly 8 per cent. of the national income.

Have we a health service so much better than when it cost so very much less? Is it necessary, is it even prudent to divert so large a part of our resources from production to the care of the sick? And even if we could afford it in money and materials (which might be increased), can we spare so many men and women—and are they available? For example: in 1937 we lacked 25,000 nurses; in 1947, 30,000, and now in 1950, 40,000! The few electors who are capable of any real thinking have indeed food for thought.

*Sir Stafford Cripps. House of Commons, March 14th, 1950.

